# Antrodia cinnamomea extract inhibits the proliferation of tamoxifen-resistant breast cancer cells through apoptosis and skp2/microRNAs pathway

**DOI:** 10.1186/s12906-018-2204-y

**Published:** 2018-05-09

**Authors:** Yu-Shih Lin, Yin-Yin Lin, Yao-Hsu Yang, Chun-Liang Lin, Feng-Che Kuan, Cheng-Nan Lu, Geng-He Chang, Ming-Shao Tsai, Cheng-Ming Hsu, Reming-Albert Yeh, Pei-Rung Yang, I-Yun Lee, Li-Hsin Shu, Yu-Ching Cheng, Hung-Te Liu, Kuan-Der Lee, De-Ching Chang, Ching-Yuan Wu

**Affiliations:** 1 Department of Pharmacy, Chiayi Chang Gung Memorial Hospital, Chiayi, Taiwan; 2 Department of Chinese Medicine, Chiayi Chang Gung Memorial Hospital, Chiayi, Taiwan; 3 School of Chinese medicine, College of Medicine, Chang Gung University, Tao-Yuan, Taiwan; 4 Departments of Nephrology, Chiayi Chang Gung Memorial Hospital, Chiayi, Taiwan; 5 Kidney and Diabetic Complications Research Team (KDCRT), Chiayi Chang Gung Memorial Hospital, Chiayi, Taiwan; 6 Department of Hematology and oncology, Chiayi Chang Gung Memorial Hospital, Chiayi, Taiwan; 7 Division of Hematology and Oncology, Department of Internal Medicine, Taipei Medical University Hospital, Taipei, Taiwan; 8 Institute of Molecular Biology, National Chung Cheng University, No.168, Sec. 1, University Rd., Minhsiung Chiayi County, 62102 Taiwan, Republic of China; 9 Division of Acupuncture and Chinese Traumatology, Department of TCM, Kaohsiung Chang Gung Memorial Hospital and Chang Gung University College of Medicine, Kaohsiung, Taiwan; 10 Department of Otolaryngology, Chang Gung Memorial Hospital, Chiayi, Taiwan; 11 Center of Excellence for Chang Gung Research Datalink, Chang Gung Memorial Hospital, Chiayi, Taiwan

**Keywords:** Breast cancer, Tamoxifen-resistant, skp2, microRNA, Antrodia cinnamomea

## Abstract

**Background:**

Breast cancer is the most common cancer in women and affects 1.38 million women worldwide per year. Antiestrogens such as tamoxifen, a selective estrogen receptor (ER) modulator, are widely used in clinics to treat ER-positive breast tumors. However, remissions of breast cancer are often followed by resistance to tamoxifen and disease relapse. Despite the increasing understanding of the resistance mechanisms, effective regimens for treating tamoxifen-resistant breast cancer are limited. Antrodia cinnamomea is a traditional medicinal mushroom native only to Taiwan. In this study, we aimed to examine in vitro effect of antrodia cinnamomea in the tamoxifen-resistant cancer.

**Methods:**

Antrodia cinnamomea was studied for its biological activity against proliferation of tamoxifen-resistant breast cancer by XTT assay. Next, the underlying mechanism was studied by flow cytometry, qPCR and Western’s blotting assay.

**Results:**

Our results revealed that the ethanol extract of antrodia cinnamomea (AC) can inhibit the growth of breast cancer cells, including MCF-7 cell and tamoxifen-resistant MCF-7 cell lines. Combination treatment with AC and 10^− 6^ M tamoxifen have the better inhibitory effect on the proliferation of tamoxifen-resistant MCF-7 cells than only AC did. AC can induce apoptosis in these breast cancer cells. Moreover, it can suppress the mRNA expression of *skp2* (S-phase kinase-associated protein 2) by increasing the expressions of miR-21-5p, miR-26-5p, and miR-30-5p in MCF-7 and tamoxifen-resistant MCF-7 cells.

**Conclusions:**

These results suggest that the ethanol extract of antrodia cinnamomea could be a novel anticancer agent in the armamentarium of tamoxifen-resistant breast cancer management. Moreover, we hope to identify additional pure compounds that could serve as promising anti-breast cancer candidates for further clinical trials.

## Background

Breast cancer is the most common cancer in women and affects 1.38 million women worldwide per year [1]. In the past 20 years, the development of new therapeutics has significantly reduced mortality rates. Antiestrogens such as tamoxifen, a selective estrogen receptor modulator, are widely used in clinics to treat estrogen receptor (ER)-positive breast tumors. An adjuvant therapy study of tamoxifen demonstrated a 40 to 50% reduction in the odds of recurrence and reduced mortality and temporary remissions in 30 to 50% of patients with metastatic disease [2]. However, remissions of breast cancer are often followed by resistance and disease relapse [3]. Previous studies have reported several mechanisms associated with resistance to tamoxifen, including altered expression of microRNA [3, 4]. Despite the increasing understanding of the resistance mechanisms, effective regimens for treating tamoxifen-resistant breast cancer are limited. Therefore, developing treatment regimens for tamoxifen-resistant breast cancer that are more effective and accompanied by minimal adverse effects remains a priority in breast cancer research.

Antrodia cinnamomea (AC) is a medical mushroom native only to Taiwan and is originally found growing in the empty rotting trunk of Cinnamomum kanehirai Hay. AC has been reported to have anti-inflammatory, hepatoprotective, antitumor, antioxidant, and immunomodulatory properties [5–11]. In the past years, several compounds, including steroid acids, triterpenoids, and polysaccharides, have been isolated from AC [10, 12–18]. Some studies have reported that the extract of AC can inhibit the growth of breast cancer cell lines [19, 20]. Moreover, antrodin C, extracted from AC can inhibit epithelial-to-mesenchymal transition and the metastasis of breast cancer cells [21]. However, the effects of AC on acquired tamoxifen-resistant breast cancer and the underlying mechanism remain unclear. In this study, we investigated the effects of the fruiting body extract of AC on tamoxifen-resistant breast cancer and identified the underlying mechanism.

## Methods

### Cell culture and treatment

MCF-7 cells (human breast cancer cell line) were obtained from the American Type Culture Collection. The MCF-7 cells were cultured in Eagle’s Minimum Essential Medium (EMEM) (Invitrogen Corp., Carlsbad, CA), supplemented with 10% FBS at 37 °C and 5% CO2. For the acquired tamoxifen resistant breast cancer cells, MCF-7 cells were cultured in Eagle’s Minimum Essential Medium containing 10% charcoal-stripped fetal bovine serum with 1 μM tamoxifen over a period of 3 months and then maintaining them in 10^− 7^ M tamoxifen for more than 9 months. Powdered fruiting bodies of 10 months cultivated dried Antrodia cinnamomea (250 g, brought form Fong Chun biotecology Co., Taiwan) were soaked in 500 ml ethanol for three days. The sample was filtered with filter paper while the residue was further extracted twice more under the same conditions. The filtrates collected from three separate extractions were combined and evaporated to dryness under vacuum (27 g). The extract was dissolved in ethanol and stored at − 20 °C. For all experiments, final concentrations of the tested compound were prepared by diluting the stock with ethanol. Antcin K, antcin C, antcin B, methyl antcinate B, eburicoic acid and dehydroeburicoic acid were kindly gifts from Dr. Lih-Geeng Chen. Human breast cancer cells were cultured to 60–70% confluence prior to treatment. Medium was then replaced with fresh medium containing AC in DMSO (dimethyl sulfoxide) at the indicated concentrations. Cells treated with DMSO alone were used as untreated controls. The parental cells without treatment were used as blank control.

### XTT assay

The indicated breast cancer cell lines were plated at a density of 10^3^ per well, in 96-well plates, in EMEM containing 10% FBS and 1% penicillin/streptomycin. Once attached, the medium was replaced with EMEM containing 10% FBS and 1% penicillin/streptomycin. The cells were then treated with indicated drugs for 24 or 48 h; and absorbance were measured using the XTT assay kit (Roche, Cat. No. 11465015001) according to the manufacturer’s instructions as described previously [22]. The XTT formazan complex was quantitatively measured at 492 nm using an ELISA reader (Bio-Rad Laboratories, Inc.).

### Flow cytometry

Human breast cancer cells (1 × 10^6^ cells) were seeded in a 100-mm plate and cultured overnight before treatment. Then, the cells were treated with control or 10 μM of indicated drugs for 48 h. Then treated cells were detected by Annexin V-FITC Apoptosis Detection Kit (Strong Biotech Corporation, Cat No.: AVK250) and Mitoscreen JC-1 kit (Mitochondrial Membrane Potential Assay, BD Biosciences: 551302) according to the manufacturer’s instructions. In brief, at the end of the incubation period, the medium was removed. The treated cells were collected after washing by cold PBS. The supernatant was removed by centrifugation and then resuspended in indicated buffer by staining at room temperature in the dark for 15 min. The stained cells were analyzed by the flow cytometer BD FACSCanto (Becton Dickinson). Apoptosis of different developmental stages were studied by gating the respective population in the Dot Plots.

### Quantitative real time PCR

Quantitative Real time PCR were performed as described previously [23, 24]. Total RNA was extracted from the breast cancer cells using the illustra™ RNAspin Mini RNA Isolation Kit (GE Healthcare, Cat. No. 25–0500) and according to the manufacturer’s instructions. Reverse transcription was performed using the Superscript first strand synthesis kit (Invitrogen, Number: 11904018). Quantitative real-time PCR analyses using the comparative CT method were performed on an ABI PRISM 7700 Sequence Detector System using the SYBR Green PCR Master Mix kit (Perkin Elmer, Applied Biosystems, Wellesley, MA, USA) according to the manufacturer’s instructions. Following initial incubation at 50 °C for 2 min and 10 min at 95 °C, amplification was performed for 40 cycles at 95 °C for 20 s, 65 °C for 20 s and 72 °C for 30 s. Primers used were: *Skp2* forward, 5′-TTA GTC GGG AGA ACT TTC CAG GTG-3′ and *Skp2* reverse, 5′-AGT CAC GTC TGG GTG CAG ATTT-3′. *RhoA* forward, 5′-GAG CAC ACA AGG CGG GAG-3′ and *RhoA* reverse, 5’-CTT GCA GAG CAG CTC TCG TAG-3′. *GAPDH* forward, 5’-TGC ACC ACC AAC TGC TTAGC-3′ and *GAPDH* reverse, 5’-GGC ATG GAC TGT GGT CATGA-3′. *GAPDH* was used as the housekeeping gene for data normalization. For microRNA, qPCR assay was performed in accordance with the previous study [25].

Total miRNA was extracted from breast cancer cells using the mirVana™ miRNA Isolation Kit (Life Technologies Corporation, Cat. No. AM1560) according to the manufacturer’s instructions. Reverse transcription was performed using TaqMan microRNA Reverse transcription kit (Life Technologies Corporation, Number: 4366596). Quantitative real-time PCR analyses using the comparative CT method were performed on an ABI PRISM 7700 Sequence Detector System using the SYBR Green PCR Master Mix kit (Perkin Elmer, Applied Biosystems, Wellesley, MA, USA) according to the manufacturer’s instructions. Following initial incubation at 50 °C for 2 min and 10 min at 95 °C, amplification was performed for 40 cycles at 95 °C for 20 s, 65 °C for 20 s and 72 °C for 30 s. Primers used were: miR-16 forward, 5′- CGC GCT AGC AGC ACG TAA AT-3′ and miR-16 reverse, 5′- GTG CAG GGT CCG AGG T-3′. miR-21-5p forward, 5′- GCC CGC TAG CTT ATC AGA CTG ATG-3′ and miR-21-5p reverse, 5′- GTG CAG GGT CCG AGG T-3′. miR-26-5p forward, 5′- CGC CGC TTC AAG TAA TTC AGG AT-3′ and miR-26-5p reverse, 5′- GTG CAG GGT CCG AGG T-3′. miR-30-5p forward, 5′- GCG TGT AAA CAT CCT CGA CTG G-3′ and miR-30-5p reverse, 5′- GCA GGG ACC GTG GT-3′. miR-16 was used as the housekeeping gene for data normalization.

### Western blot analysis

Western blot analyses were performed as described previously [26, 27]. Cellular extracts of the human breast cancer cell line treated with DMSO or indicated compounds for 24 h were prepared according to the manufacturer’s instructions. The equal amounts of protein were fractionated on a 6 or 12% SDS-PAGE and transferred to polyvinylidene difluoride (PVDF) membranes. The membranes were then blocked with 5% nonfat dried milk for 30 min and incubated in primary antibody for 3 h in room temperature. The primary antibodies used were: anti-PARP antibody (Cell Signaling, ratio: 1:1000), anti-Skp2 antibody (Cell Signaling, ratio: 1:1000), anti-β-actin antibody (Santa Cruz, IB: 1:10000). The primary antibody and secondary antibody were diluted with 1% nonfat dried milk in 1X TBST (Tris-Buffered Saline Tween-20). Blots were washed by 1X TBST and incubated in horseradish peroxidase-conjugated secondary anti-mouse or anti-rabbit antibodies (Santa Cruz, ratio: 1:5000) for one hour in room temperature. After washing by 1X TBST again, protein signal was detected by chemiluminescence, using the Super Signal substrate (Pierce, Number: 34087).

### High-performance liquid chromatography (HPLC) conditions

HPLC analysis was performed in an Agilent 1100 HPLC system using a column (Nova-Pak® C18, 4 μm, 3.9 × 150 mm). The two solvents, trifluoroacetic acid (0.05%) and CH_3_CN were the A and B solvents, respectively, for the mobile phase. Gradient elution was programmed as follows: (A)/(B) = 95/5 (0 min) → (A)/(B) = 0/100 (40 min). Chromatograms were recorded at a flow of 0.6 ml/min and at a wavelength of 254 nm with the column maintained at 25 °C.

### miRNA target prediction

The analysis of miRNA predicted targets was determined using the algorithm TargetScan (http://www.targetscan.org/vert_71/) [28].

### Statistical analyses for cell line studies

All values were the means ± standard error of mean (SEM) of the replicate samples (*n* = 3 to 6, depending on the experiment) and experiments were repeated a minimum of three times. Differences between two groups were assessed using the unpaired two-tailed Student’s *t*-test or by ANOVA if more than two groups were analyzed. The Tukey test was used as a post-hoc test in ANOVA for testing the significance of pairwise group comparisons. *P*-values < 0.05 were considered statistically significant in all comparisons. SAS9.4 was used for all calculations.

## Results

### The composition of AC

**Fig. 1:**
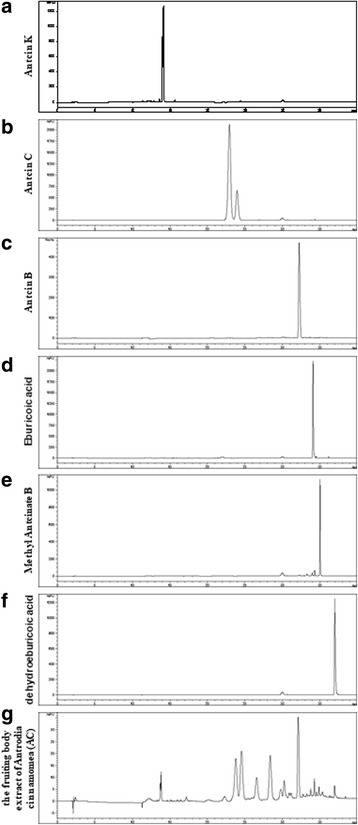
HPLC chromatograms of antcin K (**a**), antcin C (**b**), antcin B (**c**), eburicoic acid (**d**), methyl antcinate B (**e**), dehydroeburicoic acid (**f**) and the extract of Antrodia cinnamomea (AC) (**g**)

AC is a mushroom that is traditionally used for health reasons in Taiwan. Triterpenoids isolated from AC, including antcin K, antcin B, eburicoic acid, and dehydroeburicoic acid, exhibit antitumor effects [29–34]. We assessed the composition of AC through high-performance liquid chromatography (HPLC) analysis. Our HPLC result demonstrated that AC contained antcin K, antcin C, antcin B, methyl antcinate B, eburicoic acid, and dehydroeburicoic acid (Fig. 1). These data suggest that AC may achieve potential antitumor abilities due to these compounds.

### Effect of AC on the proliferation of different breast cancer cell lines

**Fig. 2:**
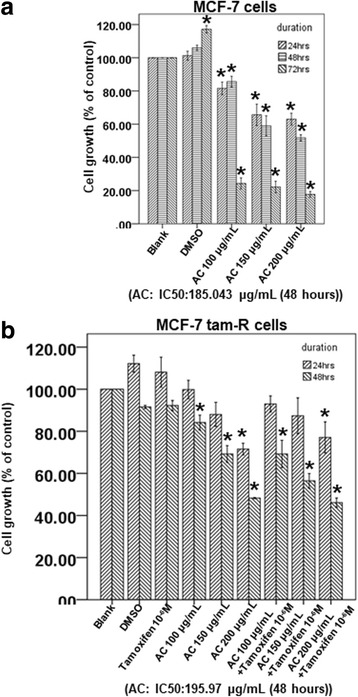
AC block the proliferation of breast cancer cell lines. MCF-7 cells **(a)** or tamoxifen resistant MCF-7 cells (MCF-7 tam-R cells) **(b)** were measured by XTT assay after indicated hours of culturing in the presence of indicated compounds. All the results are representative of at least three independent experiments. (Error bars = mean ± S.E.M. Asterisks (*) mark samples significantly different from blank group with *p* < 0.05)

To study the effects of AC on human ER-positive breast cancer cells, MCF-7 cells and acquired tamoxifen-resistant MCF-7 cells were used as a tumor cell model. First, we tested the effects of various doses of AC on the proliferation of the two breast cancer cell lines through the XTT assay. Our result revealed that AC significantly inhibited the growth of MCF-7 cells in a time- and dose-dependent manner (IC50: 185.043 μg/mL in 48 h) (Fig. 2a). Moreover, AC inhibited the proliferation of tamoxifen-resistant MCF-7 cells in a dose-dependent and time-dependent manner (IC50: 195.97 μg/mL in 48 h) (Fig. 2b). Next, we investigated that effect of combine therapy with AC and tamoxifen on the tamoxifen-resistant breast cancer cells. In Fig. 2b, 10^− 6^ M tamoxifen can not inhibit the proliferation of tamoxifen-resistant MCF-7 cells. However, combination treatment with AC and 10^− 6^ M tamoxifen can inhibit the proliferation of tamoxifen-resistant MCF-7 cells in a dose-dependent and time-dependent manner, even better than AC only did (Fig. 2b).

These data suggested that AC exhibits a potent growth inhibitory activity in human ER-positive breast cancer cells, including those resistant to tamoxifen. Combine therapy with tamoxifen have the better inhibitory effect on the proliferation of tamoxifen-resistant MCF-7 cells than only AC did.

### Effects of AC on the apoptosis of breast cancer cells in vitro

**Fig. 3:**
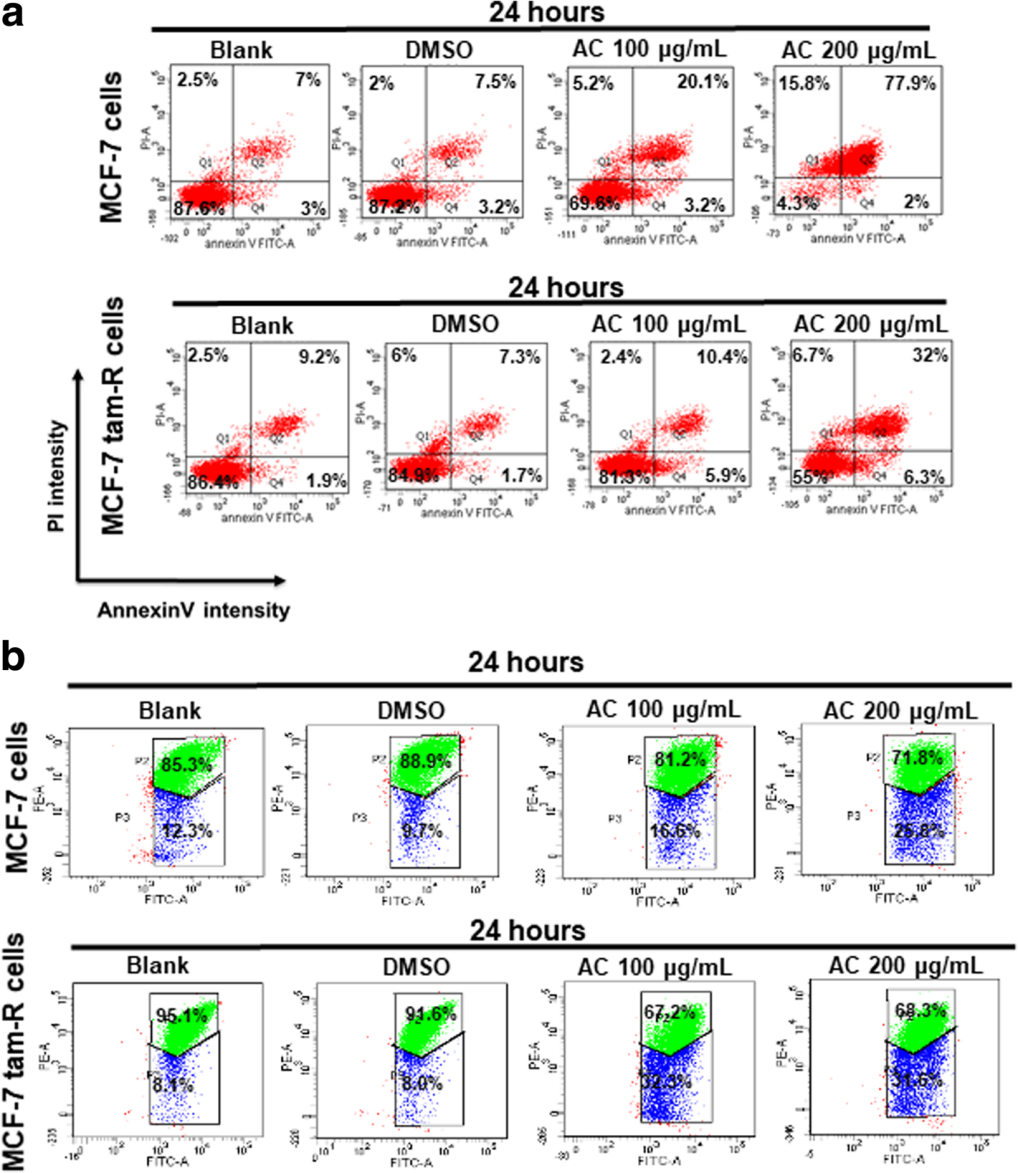
AC induces apoptosis in breast cancer cells. Breast cancer cells were treated without or with indicated compounds for 24 h. Cell apoptosis was detected by flow cytometry with annexin-V-FITC/PI dual staining or mitochondrial membrane potential assay (mitoscreen JC-1 staining assay). **a** For annexin-V-FITC/PI dual staining, the representative histograms of flow cytometric analysis using double staining with annexin-V-FITC (FITC-A) and PI (PI-A). Q1 (annexin-V−/PI+) show necrosis cells; Q2 (annexin-V+/PI+) show the late apoptosis cells; Q3 (annexin-V−/PI−) show normal cells; Q4 (annexin-V+/PI−) show the early apoptosis cells. **b** For mitochondrial membrane potential assay (mitoscreen JC-1 staining assay), dot Plots revealing depolarization of mitochondria in treated HCT 116 cells. The percentage of events in the upper gate (P2) and lower gate (P3) represent population of treated breast cancer cells having normal and depolarized mitochondria respectively

To further investigate whether the inhibition of cell proliferation induced by AC was associated with apoptosis, we determined the mechanism of AC in the two breast cancer cells. The cells were treated with the indicated compounds for 24 h, and then cell apoptosis was analyzed through flow cytometry with annexin V/PI dual staining. The results demonstrated that AC significantly induced apoptosis in the two breast cancer cells in a dose-dependent manner (Fig. 3a). Tetraethyl benzimidazolyl carbocyanine iodide (JC-1), a cationic dye, can accumulate in energized mitochondria. JC-1 exists in two different states, including aggregates (at high concentrations) or monomers (at low dye concentrations), each with the different emission spectra. At low concentrations from low mitochondrial membrane potential, JC-1 yields green fluorescence with emission of 530 ± 15 nm. At high concentrations from high mitochondrial membrane potential, JC-1 yields a red to orange colored emission of 590 ± 17.5 nm. A decrease in the aggregate fluorescent count is indicative of depolarization whereas an increase is indicative of hyperpolarization. By using the mitochondrial membrane potential assay (mitoscreen JC-1 staining assay), we also confirmed that the 2 breast cancer cells treated with AC underwent apoptosis through mitochondrial depolarization (Fig. 3b). These results suggested that apoptosis is the mechanism of cell death induced by AC in ER-positive and tamoxifen-resistant breast cancer cells.

### AC reduces the protein expression and mRNA level of skp2

**Fig. 4:**
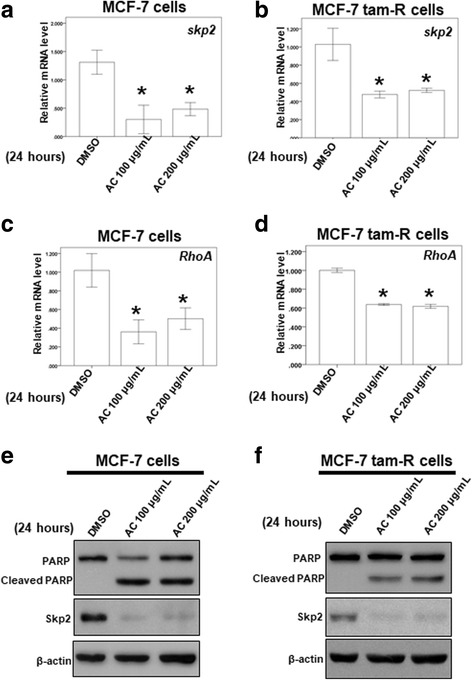
The effect of AC on the mRNA expression of *skp2* and *RhoA* and the protein expression of PARP and skp2. **a-d** Total mRNA was extracted from the two breast cancer cells after treat without or with indicated drugs for 24 h. The coding regions of human *Skp2* and *RhoA* were used as probes for real time polymerase chain reaction analysis. **e, f** Total cell extracts of the two breast cancer cells were harvested from cells treated with DMSO or indicated concentrations of AC for 24 h. The protein was immunoblotted with polyclonal antibodies specific for *PARP* or Skp2. β-actin was used as an internal loading control. (Error bars = mean ± S.E.M. Asterisks (*) mark samples significantly different from DMSO group with *p* < 0.05)

In a previous study, skp2 (S-phase kinase-associated protein 2) was significantly overexpressed in breast cancer samples and cell lines, and a high skp2 expression positively correlated with poor prognosis of breast cancer [35]. The Ras homolog gene family, member A (RhoA) GTPase is crucial for cancer metastasis, and RhoA transcription is regulated by the Skp2 complex [36]. To determine the mechanism by which AC regulates the proliferation of breast cancer cells, we also determined the mRNA and protein expression of skp2 in the 2 breast cancer cells treated with AC. Our results revealed that AC inhibited the mRNA expression of skp2 in MCF-7 and acquired tamoxifen-resistant MCF-7 cells (Fig. 4a and b). We also determined the mRNA expression of *RhoA* through qPCR. AC inhibited the mRNA expression of *RhoA* in both the breast cancer cells (Fig. 4c and d). We also found that AC inhibited the protein expression of skp2 in MCF-7 and tamoxifen-resistant MCF-7 cells. In addition, AC induced the protein expression of cleaved poly (ADP-ribose) polymerase (PARP) in both the breast cancer cell lines (Fig. 4e and f). Our data suggested that AC inhibits growth in both ER-positive and tamoxifen-resistant breast cancer cells by modulating apoptosis and the protein and mRNA expressions of skp2.

### Effect of AC on microRNAs for regulating skp2

In previous studies, microRNAs have been reported to inhibit cancer cell proliferation and induce apoptosis by targeting the skp2 pathway [37, 38]. In Fig. 4e and f, we found the protein expression of Skp2 decreased in both ER-positive and tamoxifen-resistant breast cancer cells under AC treatment. Furthermore, qPCR showed that the mRNA expression of *Skp2* and downstream genes, *RhoA*, was inhibited by treatment of both ER-positive and tamoxifen-resistant breast cancer cells with AC (Fig. 4a-d). MicroRNA may be involved in the mechanism of AC involving the skp2 signaling pathway.

**Fig. 5:**
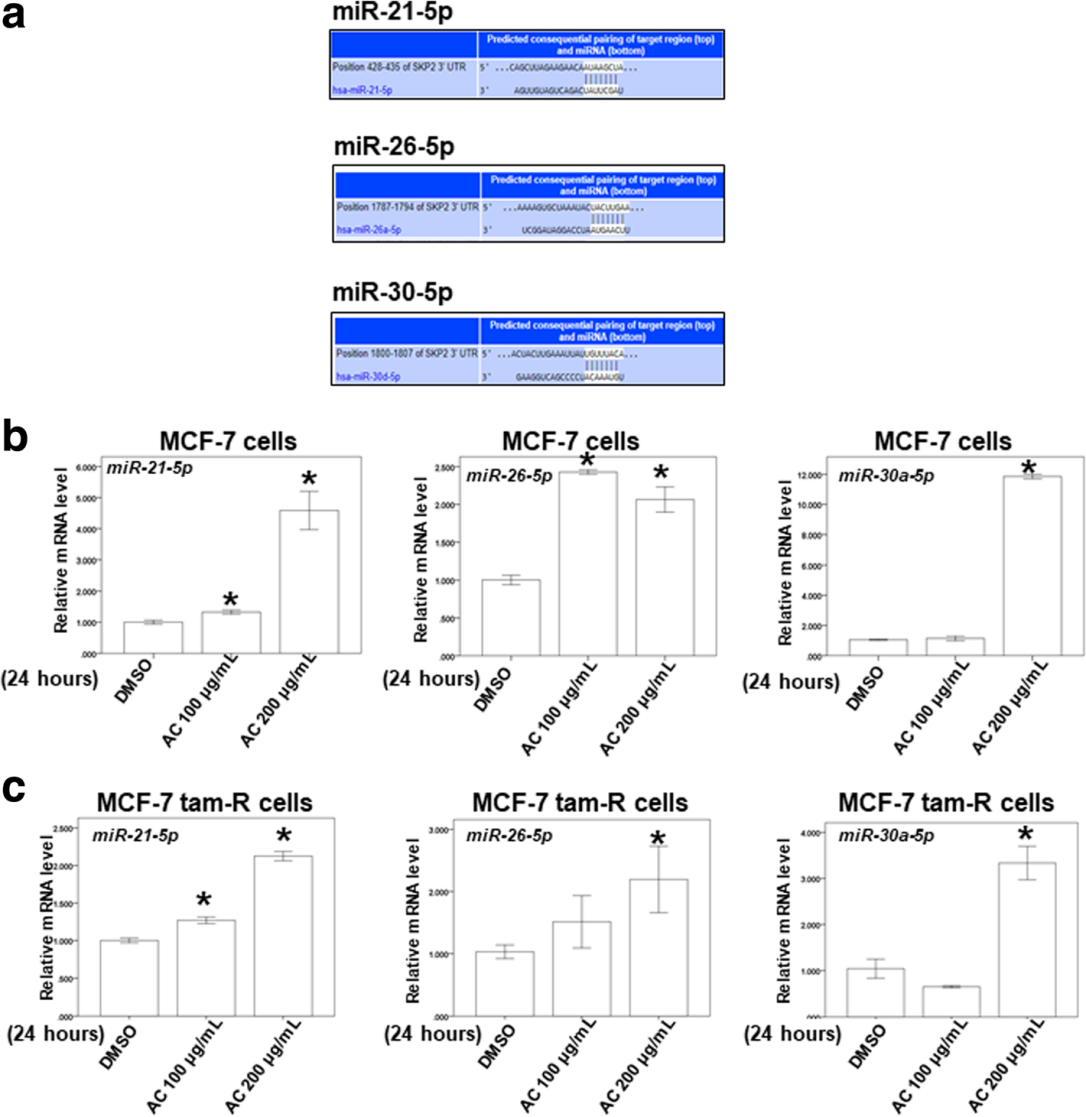
The effect of AC on the microRNA expression of miR-21-5p, miR-26-5p and miR-30a-5p. **a** miRNA target prediction of skp2. The conserved miR-21-5p, miR-26-5p and miR-30a-5p binding sites in skp2 3’-UTR region are detected by TargetScan analysis. **b-c** Total microRNA was extracted from the two breast cancer cells after treat without or with indicated drugs for 24 h. The coding regions of miR-21-5p, miR-26-5p and miR-30a-5p were used as probes for real time polymerase chain reaction analysis. (Error bars = mean ± S.E.M. Asterisks (*) mark samples significantly different from DMSO group with *p* < 0.05)

To investigate this hypothesis, a TargetScan analysis (http://www.targetscan.org/vert_71/) was applied to identify putative microRNA targets for Skp2 [28]. The TargetScan analysis revealed 3 potential microRNAs for targeting Skp2, namely miR-21-5p, miR-26-5p, and miR-30-5p (Fig. 5a). To validate this result, we determined the microRNA expression through qPCR analysis in different breast cells treated with dimethyl sulfoxide or AC. Our results revealed that AC increased the microRNA expression of miR-21-5p, miR-26-5p, and miR-30-5p in MCF-7 and tamoxifen-resistant MCF-7 cells (Fig. 5b and c). Our data suggested that AC suppressed the mRNA expression of Skp2 by increasing the expression of miR-21-5p, miR-26-5p, and miR-30-5p in both ER-positive and tamoxifen-resistant cells.

## Discussion

MCF-7 is a stable tumor cell line derived from a patient with metastatic breast cancer; it contains ERs and is estrogen responsive. Moreover, it is the most widely used and best characterized cell line for investigating acquired tamoxifen resistance [39–43]. For tamoxifen-resistant MCF-7 cells, one proteome report demonstrated that tamoxifen-resistant breast cancer cells are characterized by downregulated ER signaling, the activation of alternative survival pathways, and enhanced cell motility, including RhoA protein [39]. Our result revealed that AC exhibited a higher IC50 for tamoxifen-resistant MCF-7 cells (195.97 μg/mL) than that for MCF-7 cells (185.043 μg/mL). Thus, tamoxifen-resistant MCF-7 cells may activate some alternative survival pathways against these compounds. Moreover, AC can inhibit the mRNA expression of *RhoA*, the upregulated protein in tamoxifen-resistant breast cancer cells, in both the ER-positive and tamoxifen-resistant breast cancer cells (Fig. 4c and d). These results suggest that RhoA signaling may be an important pathway through which AC can inhibit the growth of both the ER-positive breast cancer cells and breast cancer cells with acquired tamoxifen resistance.

Some previous studies have demonstrated that AC extract can inhibit the growth of breast cancer cell lines [20, 21]. Shang et al. demonstrated that AC fruiting body extracts can deregulate the PI3K/Akt/mTOR signaling pathway and induce apoptosis in T47D breast cancer cells [20]. Kumar et al. demonstrated that antrodin C attenuates the TGF-β1-induced epithelial mesenchymal transition, migration, and invasion of MCF-7 cells through the suppression of Smad2/3 and β-catenin signaling pathways [21]. Su reported that ethyl acetate extracts of AC exhibited antiproliferation effects against MDA-MB-231 cells, a triple-negative breast cancer cell line [19]. However, the effect of AC on the acquired tamoxifen-resistant breast cancer remains unclear. Our results revealed that AC could inhibit both the ER-positive and acquired tamoxifen-resistant MCF-7 cells. Our data also suggested that AC could be developed as a potential candidate against acquired tamoxifen-resistant breast cancer.

Shang et al. used tree different fruiting bodies of AC harvested at 3, 6, and 9 months to investigate their cytotoxicity against T47D cells, a human mammary ductal carcinoma cell line (ER-positive). After 48 h of treatment, the IC50 of T47D cells was 52.7 μg/mL (3-mo fruiting body), 122.6 μg/mL (6-mo fruiting body), and 141.9 μg/mL (9-mo fruiting body) [20]. In our study, we used the 10-month fruiting body of AC; the IC50 values of tamoxifen-resistant MCF-7 and MCF-7 cells were 195.97 and 185.043 μg/mL, respectively. The difference between these results may be attributed to the different culture duration and different breast cancer cell lines. Our HPLC result revealed that the composites of AC included antcin K, antcin C, antcin B, methyl antcinate B, eburicoic acid, and dehydroeburicoic acid. In previous studies, antcin K has been shown to exhibit antitumor effects against hepatoma and leukemia [12, 29, 44]. Antcin C, antcin B, and eburicoic acid also exhibit antitumor effects against hepatoma [31, 45, 46]. Dehydroeburicoic acid exhibits antitumor effects against the breast cancer T47D cell line [20]. Thus, dehydroeburicoic acid in AC may play an important role against breast cancer. However, numerous composites in AC remain unidentified. These composites also play an important role in the antitumor activity against breast cancer, including ER-positive and acquired tamoxifen-resistant breast cancer.

Bhatt et al. demonstrated tamoxifen can stimulate p38MAPK (p38 mitogen-activated protein kinases) to catalyze phosphorylation of Skp2 at serine-64 that drives ubiquitin-dependent proteasomal degradation of NKX3–1 to induce Oct-4 (octamer-binding transcription factor 4) gene expression in the presence of tamoxifen. These results suggested the crucial role of Skp2/p38MAPK/NKX3–1 mediated Oct-4 expression in driving tamoxifen resistance in MCF-7 cells [47]. Previous studies have demonstrated that microRNAs such as miR-186 and miR-340 can inhibit cancer cell proliferation and induce apoptosis by targeting the Skp2 pathway [37, 38]. Another previous study demonstrated that AC exerts an inhibitory effect on breast cancer stem cells from the MDA-MB231 cell line. AC also downregulated several microRNAs, including miR-381 and miR-711, in MDA-MB231 cells, a triple-negative breast cancer cell line [19]. However, the effect of AC on the expression of microRNA and Skp2 in ER-positive breast cancer and acquired tamoxifen-resistant breast cancer remain unclear. We first demonstrated that AC suppresses the mRNA expression of skp2 by increasing the expression of miR-21-5p, miR-26-5p, and miR-30-5p in MCF-7 and tamoxifen-resistant MCF-7 cells. In previous study, higher levels of miR-26a were significantly associated with clinical benefit and prolong time to progression on tamoxifen therapy of breast cancer [48]. Higher expression levels of miR-30 family (miR-30a and miR-30c) were significantly associated with benefit of tamoxifen treatment and with longer progression free survival of breast cancer patients [49]. Our data suggested AC inhibited different breast cancer cell lines through different microRNA signaling pathways. It is possible that AC may have clinical benefit of tamoxifen treatment in breast cancer patients. However, AC is the crude extract and contain a lot of pure compounds, many different mechanisms are involved through these different pure compounds [10, 12–18]. It is possible that Skp2 signaling pathway is not the only major pathway under the treatment of AC. In the further, we will investigate the in vivo and in vitro effect of these pure compounds on the breast cancer to clarify the clear mechanism of pure compounds with respect to tamoxifen resistance.

## Conclusion

In summary, this is a novel study that demonstrated the mechanism by which AC can inhibit the proliferation of ER-positive and acquired tamoxifen-resistant breast cancer cells. The study results suggest that AC could be a novel anticancer agent in the armamentarium of breast cancer management. Furthermore, additional pure compounds from AC that might serve as promising anti-breast cancer candidates should be identified for further clinical trials.
